# Sex chromosomes in the tribe *Cyprichromini* (Teleostei: Cichlidae) of Lake Tanganyika

**DOI:** 10.1038/s41598-022-23017-y

**Published:** 2022-10-26

**Authors:** Kristen A. Behrens, Stephan Koblmüller, Thomas D. Kocher

**Affiliations:** 1 Department of Biology, University of Maryland, College Park, MD 20742 USA; 2 Institute of Biology, University of Graz, Universitätsplatz 2, 8010 Graz, Austria

**Keywords:** Computational biology and bioinformatics, Evolution, Genetics

## Abstract

Sex determining loci have been described on at least 12 of 22 chromosomes in East African cichlid fishes, indicating a high rate of sex chromosome turnover. To better understand the rates and patterns of sex chromosome replacement, we used new methods to characterize the sex chromosomes of the cichlid tribe *Cyprichromini* from Lake Tanganyika. Our k-mer based methods successfully identified sex-linked polymorphisms without the need for a reference genome. We confirm the three previously reported sex chromosomes in this group. We determined the polarity of the sex chromosome turnover on LG05 in *Cyprichromis* as ZW to XY. We identified a new ZW locus on LG04 in *Paracyprichromis brieni.* The LG15 XY locus in *Paracyprichromis nigripinnis* was not found in other *Paracyprichromis* species, and the sample of *Paracyprichromis sp. “tembwe*” is likely to be of hybrid origin. Although highly divergent sex chromosomes are thought to develop in a stepwise manner, we show two cases (LG05-ZW and LG05-XY) in which the region of differentiation encompasses most of the chromosome, but appears to have arisen in a single step. This study expands our understanding of sex chromosome evolution in the Cyprichromini, and indicates an even higher level of sex chromosome turnover than previously thought.

## Introduction

Sex chromosomes often arise from autosomes when a new mutation alters the function of developmental pathways in the gonad^1,2^. While the original mutation may be as small as a single-nucleotide variant (SNP), sex chromosomes often show substantial sequence divergence over regions of several million base pairs. Theory suggests that this region of differentiation arises because selection favors a reduction in recombination around the novel sex mutation, in order to maintain linkage between the sex locus and sexually antagonistic variants at nearby loci^3^. Two pathways for the evolution of these divergent sex chromosomes have been proposed^4^. The first is a gradual extension of the region of reduced recombination via a mechanism of recombination suppression that is spatially continuous in its effects. The second is a stepwise expansion in which inversions or other structural mutations instantly restrict recombination across large blocks of the chromosome, leading to the formation of distinct evolutionary strata^5,6^. Once recombination between the new sex chromosome and its homolog has been reduced, the non-recombining chromosome will accumulate deleterious mutations and degenerate via the Hill-Robertson effect^2,7,8^. The rates and patterns of sex chromosome degeneration currently are not well understood^9^.

New autosomes can be recruited to become sex chromosomes, causing transitions from one sex chromosome system to another. Sex chromosomes may also evolve through chromosome fusions, which have been documented to occur frequently in fish, or via a transposition of a sex determiner to a new chromosome^10^. We distinguish homogeneous transitions (e.g., XY to XY) from heterogeneous transitions (e.g., XY to ZW). Transitions can involve a single chromosome (i.e. cis), or can involve transition to a new chromosome (trans)^11^. When transitions occur in cis, heterogeneous transitions are easier to detect than homogeneous transitions^12^. Several mechanisms have been proposed to explain the occurrence of sex chromosomes transitions. The mutational load theory proposes that sex chromosomes may be replaced when too many deleterious mutations accumulate on the non-recombining chromosome^12,13^. An alternative theory is that new sex loci are recruited to resolve genetic conflicts^5,14–16^. New sex chromosomes may also invade via genetic drift^17^ or selection on sex ratio^18,19^.

Old sex chromosome systems can often be detected cytologically as differences in the size or structure of the sex chromosome pair. In contrast, young sex chromosomes can be challenging to identify cytologically because the non-recombining region may be small or not highly differentiated. Sequence-based methods for detecting sex chromosomes for detecting younger sex chromosomes have improved with each new DNA sequencing technology by decreasing sequencing cost, and improving the quality of reference genome sequences (reviewed in Palmer et al.^20^). Genome wide association studies (GWAS) easily detect many sex chromosome systems, but analysis of quantitative trait loci (QTL) in families may be needed when the sex chromosomes are very young^21^. Recently, methods analyzing the frequency of k-mers in DNA sequences have come to the forefront^22,23^. K-mer analysis can identify structural variants in addition to SNPs, and can identify sex-linked regions even in the absence of a reference genome^24^.

The great diversity and frequent turnover of sex chromosomes among fish species is an opportunity to better understand the rates, patterns, and mechanisms of sex chromosome evolution^10,25^. Only a small subset of fish (~ 10%) feature heteromorphic sex chromosomes that can be distinguished by cytogenetic methods^26,27^. In this paper XY and ZW will refer to male and female heterogamety, respectively, regardless of whether the sex chromosomes can be distinguished cytologically. For most species, the characterization of sex chromosomes must be done by analyzing DNA sequences. East African cichlid fishes (Cichlidae) have been the subject of extensive research on sex chromosome evolution, due to their great diversity and rapid radiation^28^. Sex determining loci have been described on at least 12 of 22 chromosomes in cichlids to date, with additional sex determiners found on B chromosomes^11,29–31^. Turnover of sex chromosomes in cichlids is also high, estimated to be 0.187 turnovers per million years compared to 0.02 turnovers per million years estimated for ricefishes^32^.

The Cyprichromini are a tribe of small shoaling planktivorous cichlids from Lake Tanganyika that often occur in large mixed-species schools^33,34^ and share a common ancestor about 4 MYA^35,36^. There are two genera, *Cyprichromis* and *Paracyprichromis,* that include seven species that have been formally described, and at least five additional forms that likely deserve species status^32^. Previous work has identified sex chromosomes in 8 of the 12 species, including a ZW system on LG05 in *Cyprichromis leptosoma*^37^, *C.* sp. “dwarf jumbo” and *C. coloratus*, XY systems on LG05 in *Cyprichromis microlepidotus*, *Cyprichromis pavo*, *Cyprichromis zonatus*, and *Cyprichromis* sp. "kibishi”, and an XY system on LG15 in *Paracyprichromis nigripinnis*^32^*.* The close evolutionary relationships of these species make the tribe *Cyprichromini* a useful system for studying transitions among sex chromosome systems and the development of sequence divergence in the early stages of sex chromosome evolution. Of particular interest is the putative *cis* heterogeneous transition from an XY to a ZW system within this clade. Here, we employ a custom k-mer pipeline alongside traditional sex-specific SNP methods to further investigate the sex chromosomes of the tribe *Cyprichromini*.

## Methods

### Animal use

All animal experiments using live fish were conducted in accordance with the University of Maryland Institutional Animal Care and Use Committee (IACUC) protocol #R-OCT-19-48 and the Guide for Care and Use of Laboratory Animals. *Paracyprichromis* sp. “brieni south” individuals were wild caught in Zambia, and their use was approved under the University of Graz Zambian research permit K-4335/18 KA/K.48/18. This study was reported in accordance with ARRIVE guidelines.

### DNA samples and sequencing

*Paracyprichromis* sp. “brieni south” were collected from the wild near the Kalambo Lodge, Mpulungu, Zambia. We sampled tissue from 26 males and 27 females. DNA was extracted from fin clips by phenol–chloroform using phase-lock gel tubes (5Prime, Gaithersburg, MD). DNA concentrations were quantified by fluorescence spectroscopy using a Quant-iT PicoGreen assay (ThermoFisher, Waltham MA, USA). Equimolar amounts of DNA from each individual were then pooled by sex for each species. Sequencing libraries were constructed, and 150 bp paired-end DNA sequencing was performed on a NovaSeq6000 S4 (Illumina, San Diego CA) by Novogene US (Davis, CA).

### Analysis of existing sequences

Sequence data from previous studies were downloaded from the NCBI Short Read Archive and GenBank. These included genomic data from a pool-seq analysis of *C. leptosoma*^37^, genomic data from single males and females of eleven additional species^36^ and transcriptome data for two species^32^ (Table 1). Due to the high quality of the sequencing reads, a trimming step was deemed unnecessary.

**Table 1:** Samples used, * pooled, + transcriptome.

Species	Sex	# Individuals	Mean coverage	Collection location	Project Number
*Paracyprichromis* sp. "tembwe"	F	1	16.05	Tembwe DRC	PRJNA550295
*Paracyprichromis* sp. "tembwe"	M	1	16.84	Tembwe DRC	PRJNA550295
*Paracyprichromis nigripinnis*	F	1	6.45	Chituta	PRJNA550295
*Paracyprichromis nigripinnis*	M	1	12.88	Chituta	PRJNA550295
*Paracyprichromis nigripinnis* +	F	3	Unknown	N/A	PRJNA552202
*Paracyprichromis nigripinnis* +	M	3	Unknown	N/A	PRJNA552202
*Paracyprichromis* sp. "brieni south"	F	1	5.99	Kalambo Lodge, Mpulungu, Zambia	PRJNA550295
*Paracyprichromis* sp. "brieni south"	M	1	8.27	Kalambo Lodge, Mpulungu, Zambia	PRJNA550295
*Paracyprichromis brieni*	F	1	6.03	Nyaruhongoka 2	PRJNA550295
*Paracyprichromis brieni*	M	1	7.1	Nyaruhongoka 2	PRJNA550295
*Paracyprichromis* sp. “brieni south” *	F	27		Kalambo Lodge, Mpulungu, Zambia	PRJNA802233
*Paracyprichromis* sp. “brieni south” *	M	26		Kalambo Lodge, Mpulungu, Zambia	PRJNA802233
*Cyprichromis pavo*	F	1	7.66	Misepa	PRJNA550295
*Cyprichromis pavo*	M	1	9.01	Misepa	PRJNA550295
*Cyprichromis microlepidotus*	F	1	6.7	Nyaruhongoka 2	PRJNA550295
*Cyprichromis microlepidotus*	M	1	6.1	Nyaruhongoka 2	PRJNA550295
*Cyprichromis zonatus*	F	1	6.35	Chituta	PRJNA550295
*Cyprichromis zonatus*	M	1	9.79	Chituta	PRJNA550295
*Cyprichromis* sp. "kibishi"	F	1	7.33	Milila	PRJNA550295
*Cyprichromis* sp. "kibishi"	M	1	6.63	Milila	PRJNA550295
*Cyprichromis leptosoma*	F	1	8.08	Kalambo Lodge, Mpulungu, Zambia	PRJNA550295
*Cyprichromis leptosoma*	M	1	8.9	Kalambo Lodge, Mpulungu, Zambia	PRJNA550295
*Cyprichromis leptosoma* *	F	30	19.25	Kalambo Lodge, Mpulungu, Zambia	PRJNA400462
*Cyprichromis leptosoma* *	M	26	10.76	Kalambo Lodge, Mpulungu, Zambia	PRJNA400462
*Cyprichromis leptosoma* +	F	3	N/A	N/A	PRJNA552202
*Cyprichromis leptosoma* +	M	3	N/A	N/A	PRJNA552202
*Cyprichromis* sp. "dwarf jumbo"	F	1	10.64	Cave Kigoma	PRJNA550295
*Cyprichromis* sp. "dwarf jumbo"	M	1	12.84	Cave Kigoma	PRJNA550295
*Cyprichromis coloratus*	F	1	14.4	Chitweshiba	PRJNA550295
*Cyprichromis coloratus*	M	1	15.66	Chitweshiba	PRJNA550295

### Sex-specific SNP analysis

The sequence reads were aligned to the Nile tilapia (*Oreochromis niloticus—*UMD_NMBU, RefSeq GCF_001858055.2, ~ 80% properly paired) and the Malawi zebra (*Maylandia zebra*—UMD2a, RefSeq GCF_000238955.4, ~ 81–82% properly paired) assemblies^38,39^ with BWA version 0.7.12 using the default parameters along with read group labels^40^. The alignments were sorted, marked for duplicates, and indexed using Picard version 1.119 (http://broadinstitute.github.io/picard/). Alignments were then converted into an mpileup file using Samtools version 0.1.18^41^ and subsequently into a sync file using Popoolation2^42^. Base calls with a PHRED score less than 20 were filtered out of the data set. We then used Sex_SNP_finder_GA.pl (https://github.com/Gammerdinger/sex-SNP-finder) to calculate *F*_ST_ between the male and female pools or single individuals, and identify both XY and ZW patterned SNPs^43^. The results were plotted using R^44^. Read mappings in candidate regions were examined in IGV^45^.

Bedtools^46^ *make windows* and *coverage* were used to calculate the density of sex-patterned SNPs per 100kbp window. The log_2_(XY:ZW) ratio of SNP density was then calculated for each window^47^. The Kruskal–Wallis test on the ranked data was conducted in R to determine if the log ratio differed among chromosomes with Benjamini–Hochberg correction for multiple tests. If the differences were statistically significant, the Dunn’s test from the *rstatix* R package was conducted post-hoc to determine which chromosomes significantly differed from each other. The median rank of each chromosome over the 7,621 windows of the *M. zebra* UMD2a reference genome was calculated using the rank function in R, using the “first” option to resolve ties.

### k-mer analysis

We used Jellyfish v. 2.2.7^48^ to analyze the k-mer content of the sequence reads. The *count* command was used to generate lists of k-mers for each sex. At the *dump* command, a cutoff of a minimum of two occurrences per k-mer was applied using the -L 2 flag to exclude single-occurrence k-mers that may be the result of sequencing errors, and the -C flag was used to save only canonical k-mers. Due to the low sequencing coverage for some samples (average = 9.989x, range = 6.1–16.84x), we did not apply a higher stringency to avoid disposing of k-mers of interest. After testing of k-mers ranging from 21 to 31 bp we found that a k-mer size of 22 bp was best suited for this type of low coverage data. This k-mer size optimizes the number of k-mers called while also reducing noise from sequencing errors that would occur with a larger k-mer size. Additionally, a larger k-mer size might be problematic for comparisons against other species due to the potential for species-specific SNPs. K-mers from each sex were sorted lexicographically and then compared to retrieve lists of k-mers that occurred in only one sex or the other using a custom python script. The structure of this script enables us to identify sex-specific k-mers in both sexes for species in which the sex determination system is unknown.

From the male and female file for each species, one million k-mers were randomly selected and mapped against the *M. zebra* reference genome (M_zebra_UMD2a) using blast-plus v. 2.9.0 (blastn -task blastn-short -dust no -evalue 1e−3 -max_target_seqs 1 -outfmt 6). The BLAST results were used to quantify the occurrence of k-mers on each linkage group (LG) (Supplemental Fig. 1). This count was then normalized by linkage group size (bp). *Maylandia zebra* was chosen as the reference genome because it is the most closely related high quality assembly. The use of a more contiguous, but also more distant genome, such as that of *O. niloticus*^38^ would have resulted in a loss of significant data from more divergent regions. Sex chromosomes were identified when one sex featured a larger number of k-mers on a linkage group.

The core region of sex differentiation for each shared system was identified using a custom python script that generates a list of k-mers shared by all species with that system. This resulted in two core regions, one for the XY species *C. pavo*, *C. microlepidotus*, *C. zonatus*, and *C.* sp. “kibishi”, and one for the ZW system made up of *C. leptosoma*, *C.* sp. “dwarf jumbo”, and *C. coloratus*. These k-mers were then plotted on the reference genome to determine the span of the non-recombining region containing the sex determining gene, henceforth termed the sex determining region. Reads associated with the core region of the XY LG05 system were identified in the BLAST results, assembled with minia v. 0.0.102^49^, aligned to *M. zebra* with bwa v. 0.7.17^40^, and visualized in IGV. Samtools v. 1.10 was used for any necessary file format conversions, sorting, or indexing. Exact boundaries of the LG05 core regions were defined based on positional information from IGV.

## Results

### A comparison of methods in *C. leptosoma*

We previously published pool-seq data for 26 male and 30 female *Cyprichromis leptosoma*, which has a ZW sex chromosome system on linkage groups 5 and 13^37^. Transcriptome data for 3 males and 3 females is also available^50^, along with genomic data for a single male and female^32^. We used these datasets to validate and compare our several analytical methods.

**Figure 1:**
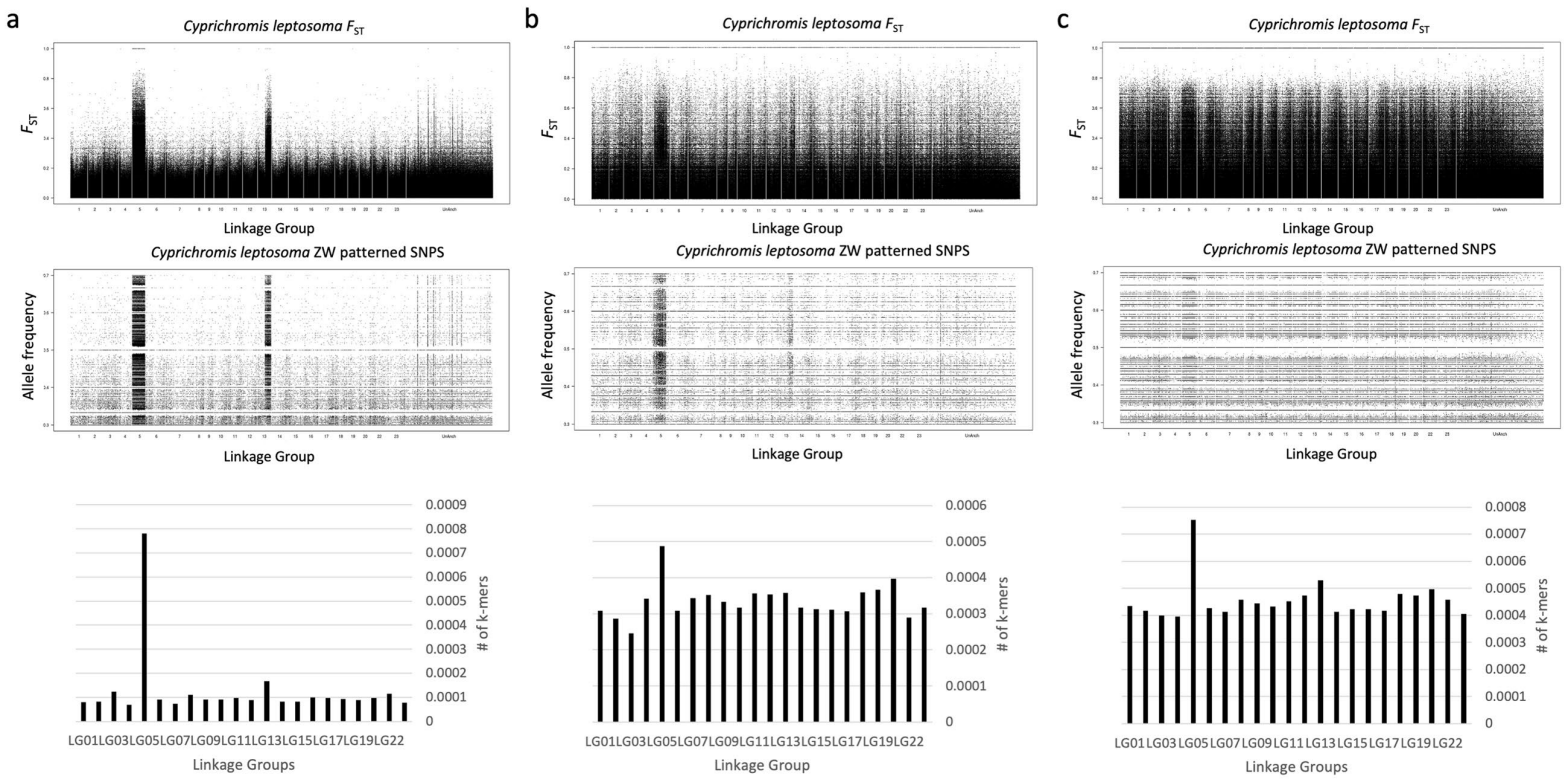
Comparison of methods in *C. leptosoma.* Top panel is the *F*_ST_ statistic, middle panel are the ZW-patterned SNPs, and the bottom panel represents k-mer counts per linkage group are normalized to chromosome size. (**a**) Genomic 26M and 30F, (**b**) Transcriptomic 3M and 3F, (**c**) Genomic 1M and 1F.

Using the genomic data from 26 males and 30 females we were easily able to identify a strong signal on LG05 and a weaker signal on LG13 using either our sex-specific SNP or k-mer methods (Fig. 1a). Our results from the k-mer pipeline are directly comparable to the sex-specific SNP method from the Gammerdinger et al.^37^ study.

The comparison of 100 kb windows for the ratio of XY to ZW SNPs revealed significant heterogeneity among chromosomes (Kruskal–Wallis, p = 2.2e−16). The post-hoc Dunn’s test showed highly significant differences between LG05, LG13, LG03 and LG11 from the other chromosomes (Supplemental Table 1). The LG05, LG13, and LG03 signals were supported by their distinct median rank from the other chromosomes, however LG11 was not, marking it as a possibly small region of differentiation or a spurious signal (Fig. 4). LG05 is the most divergent, followed by LG13, and then LG03. The signal on LG03 has not been previously detected. The strength of the significance in the Dunn’s test and median rank suggests that perhaps it is a young sex chromosome not yet detectable with other methods, or that it represents a sex chromosome segregating within the population at a lower frequency.

We also pooled the transcriptome data for 3 males and 3 females reported previously^32^. The signal for LG05 is easily observed from just this small sample of individuals (Fig. 1b), however it is clear that more individuals are required to clearly detect signal on LG13 and LG03.

When we analyzed the genomic data for single individuals, the signals for LG05 were still strong, but the LG13 peak was much less evident (Fig. 1c). The sex SNP pipeline and log_2_(XY:ZW) SNPs per 100 kb window comparison with a Kruskal–Wallis test still yielded a significant result (p-value = 2.2e−16). The Dunn’s test showed highly significant differences between LG05, LG13, and to a lesser extent LG07 and LG04, and the other chromosomes (Supplemental Table 1). The LG05 and LG13 signals were confirmed by their distinct median rank from the other chromosomes (Fig. 4). Signals on LG07 and LG04 are not apparent on the median rank plot, so we can conclude that they are spurious signals resulting from polymorphisms in the single individual samples. Thus, it appears that this k-mer method can be applied to detect sex chromosomes in other species. However, young sex chromosomes may be difficult to detect with any method, as evidenced by the fact that the LG03 signal was not strongly evident with the k-mer method.

### LG05-ZW systems in other *Cyprichromis*

We next applied our k-mer analysis to the ZW systems on LG05 in *C.* sp. “dwarf jumbo” and *C. coloratus* identified from single individuals of each sex^32^. The *C.* sp. “jumbo” sequenced by Ronco et al. (2021)^36^ could not be analyzed due to a lack of a corresponding female. The W chromosome of the ZW system on LG05 is highly differentiated (Fig. 3), with the sex determining region covering approximately 77% of the chromosome. The shared core region identified for the ZW group of species (*C. leptosoma, coloratus* and sp. “dwarf jumbo”) contained approximately one million sex-specific k-mers, corresponding to roughly 45,000 SNPs. There are many more female-specific (W) k-mers than male-specific (Y) k-mers. The XY-patterned k-mers likely correspond to sequences that are either absent on the W, or so highly diverged that they do not map to the reference genome. Thus, males could be heterozygous, while females would appear homozygous at these sites. These species did not show any ZW differentiation on LG13, indicating that the LG13 sex determining region is unique to *C. leptosoma*. Whether this region is the result of a fusion event or is segregating separately in the population cannot be determined without additional data.

Plotting the k-mers by position reveals that the W-specific regions encompass most of the chromosome. Potential candidate genes for sex determination include *wnt4*^37^, *ipk61*, *appl1, grip2, mst1,* and *rspo4*. Additional candidates have been suggested from the overlapping LG05 XY sex determining region in *Astatotilapia burtoni*, including *rspo4*^51^. With such a large region of differentiation, identifying a causal gene is not currently possible.

### LG05-XY systems in *Cyprichromis*

**Figure 2:**
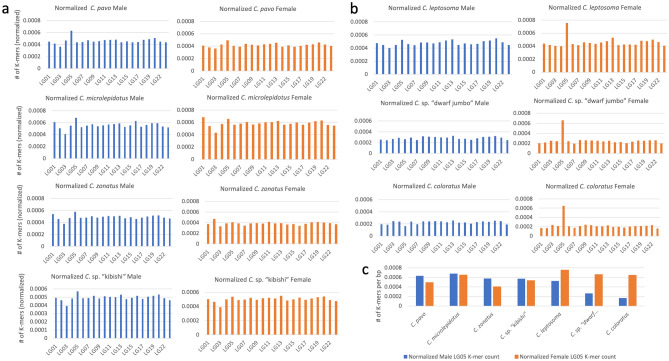
(**a**) Bar plots showing peaks on respective linkage groups called with the k-mer method from single individuals for the LG05 XY species and (**b**) for the LG05 ZW species. (**c**) side-by-side bar plot comparison of M/F k-mer counts for each species showing differentiation on LG05.

Several other species of *Cyprichromis* appear to have an XY system on LG05. A LG05 XY system in *C. microlepidotus* was previously reported from transcriptome data^32^*.* These authors further suggested XY systems on LG05 in *C. pavo*, *C. zonatus*, and *C.* sp. “kibishi” based on their tribe-level GWAS of genomic data. Our k-mer approach identified this system from the genomic data of single male/female pairs (Fig. 2)*.*

**Figure 3:**
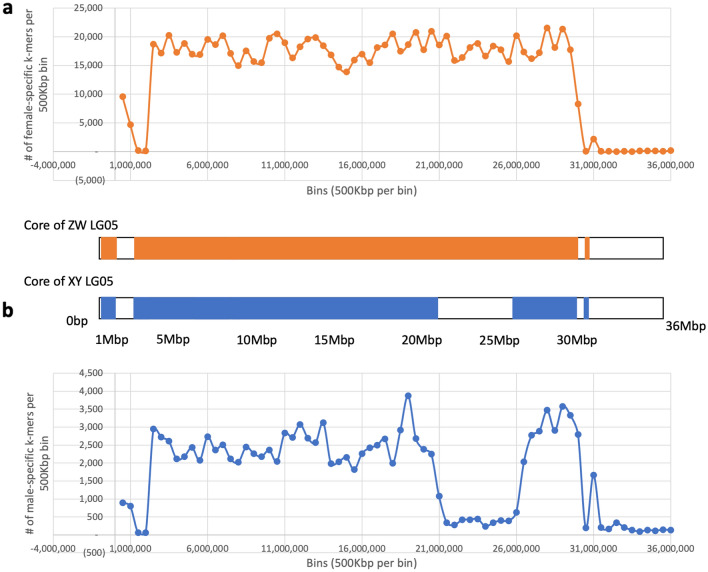
Representation of the core regions of high differentiation on LG05. (**a**) W k-mer density and conceptual representation of the regions shared between all species with a ZW system on LG05, (**b**) Y k-mer density and core region shared by the species with an XY system on LG05.

The Y chromosome of this system is highly differentiated. The core region shared by all species contains ~ 100,000 k-mers or roughly 4,500 SNPs. The total of the differentiated regions covers approximately 63% of the chromosome (Fig. 3). The region of differentiation largely corresponds to that of the ZW core region, but it lacks differentiation from 20.7 to 26.2 Mb (Fig. 3). The lack of contiguity of the differentiated regions likely reflects structural rearrangements relative to the reference genome assembly. Due to the large size of the core sex determining region, it is not possible to identify the sex determining gene, however there are numerous candidates that have the potential to fill this role. There is a large region in common between the LG05 sex determining regions in *A. burtoni* and *Cyprichromis*, spanning from approximately 14 Mbp to 17 Mbp, but the exact boundaries of the sex-determining region in *A. burtoni* have not been defined^30,51^.

A previous study suggested the ZW signal in *C. leptosoma* and the XY signal in *C. microlepidotus* were of similar magnitude^32^. However, in our analysis, although the signal in *C. microlepidotus* was clear enough to call the LG05 sex chromosome, it was much weaker than the *C. leptosoma* signal. This seems to be broadly true across *Cyprichromini* species, where the ZW systems tend to have a stronger sex-specific k-mer signal and more W-specific k-mers than the XY systems. This difference in signal suggests different levels of differentiation. The ability to distinguish the level of differentiation between the two systems allowed us to consider differences in the age of the two systems.

### Similarities among X, Y, Z and W chromosomes in *Cyprichromis*

The number of sex-specific k-mers (Fig. 3) suggests the ZW system (mean = 14,000 k-mers, median = 17,000 k-mers per 100 kb window) is approximately 7 × more differentiated than the XY system (mean = 1,700 k-mers, median = 2,000 k-mers per window). A comparison of shared male and female-specific k-mer between species with the XY and ZW system showed that the greatest number of shared LG05 k-mers was between the female-specific (X) k-mers from the XY species and the female-specific (W) k-mers from the ZW species (~ 400–700 k k-mers) (Table 2). The second greatest number of shared LG05 k-mers was between the male-specific (Y) k-mers from the XY species and the female-specific (W) k-mers from the ZW species (~ 100–200 K k-mers). We therefore suggest the ZW system is ancestral, and that a transition to an XY system occurred in which the Y arose on a W chromosome, and the remaining W chromosomes came to function as an X.

**Table 2:** Approximate number of k-mers shared between *C. leptosoma* vs. *C. microlepidotus* on LG05.

	*C. microlepidotus*	
	X (female)	Y (male)
*C. leptosoma*		
Z (male)	22,000	20,000
W (female)	686,000	181,000

### Analysis of *Paracyprichromis nigripinnis*

**Figure 4:**
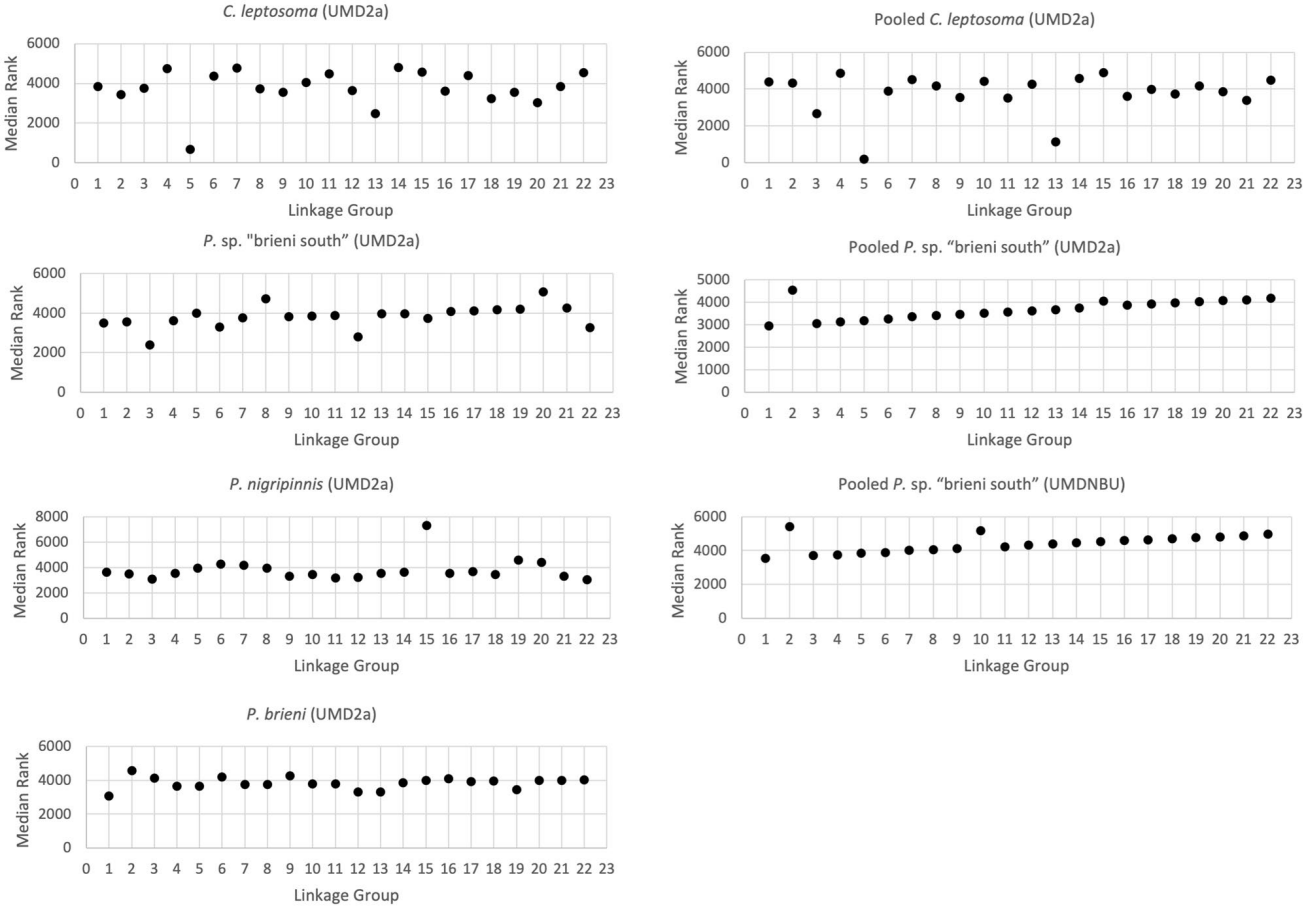
Median ranks of log_2_(XY:ZW) SNPs per 100 kb by LG for pooled and single individual data on either the UMD2a or UMDNMBU reference assemblies. Points that are high outliers suggest an XY system, points that are low outliers suggest a ZW system.

Previous work identified an excess of XY-patterned SNPs on LG15 in *P. nigripinnis*^32^. We were not able to detect this signal with the k-mer method in either the genomic or transcriptomic data, but we were able to detect it by examining sex-specific SNP density for the single individual genomic data. The comparison of 100 kb windows for the ratio of XY to ZW SNPs yielded a significant result (Kruskal–Wallis p = 2.2e−16). The pairwise Dunn’s tests showed that both LG15 and LG19 had a significantly higher ratio of XY SNPs compared to ZW SNPs than the other chromosomes (Supplemental Table 7). The LG15 signal was confirmed by its distinct median rank from the other chromosomes (Fig. 4). The signal on LG19 has not been previously detected and was not clearly different on the median rank plot. The strength of the significance in the Dunn’s test suggests that LG19 is a young sex chromosome, or a new evolutionary stratum, that is not yet detectable with other methods.

### Analysis of *Paracyprichromis* sp. “tembwe”

The k-mer approach did not identify a sex chromosome for this species, so we employed the SNP density approach. In IGV, we identified large blocks with a very high density of either XY or ZW SNPs (Supplemental Fig. 2). This suggests that the male, and probably the female, might be early generation interspecific hybrids. Thus, we did not pursue any further analyses on the individuals from this population.

### Analysis of *Paracyprichromis* sp. “brieni south”

On the tilapia reference assembly, the whole genome *F*_ST_ plot of the pool-seq data identifies a possible ZW system on LG04, with no significant signals on any other chromosome (Supplemental Fig. 3). The single chromosome plot shows that the differentiation spans ~ 30 kb on LG04 (9.21–9.24 Mb, Supplemental Fig. 4). This differentiation is not seen in the single individual data from the same locality^32^. On the *M. zebra* reference assembly, the homologous region is distributed over several unanchored scaffolds*.* There are three annotated genes in the region. *jpt2/hn1l* is an NAADP-binding protein that interacts with the ryanodine receptor to activate Ca^++^ release from the endoplasmic reticulum^52,53^. *jpt2* is highly expressed in both ovary and testis^54^, but a relationship to sex determination is not clear. *znhit1* is a subunit of the SRCAP chromatin remodeling complex and is essential to the initiation of meiosis^55^. *znhit1* is highly expressed in testis, and to a lesser extent in ovary^54^. Finally, the Ensembl annotation indicates a lncRNA at 9.24 Mb, about which little else is known.

The comparison of 100 kb windows for the ratio of XY to ZW SNPs yielded a significant Kruskal–Wallis test (p = 1.301e−11). The Dunn’s test showed significant excesses of signal on several chromosomes (LG02, LG06, LG08, Supplemental Table 3), but none of the median ranks were distinct (Fig. 4). There is no evidence that *P.* sp. “brieni south” has the LG15 XY system found in *P. nigripinnis*. The ZW differentiation on LG04 likely spans a region too small to be detected by the Dunn’s test, which is performed at the chromosome level. Further investigation is needed, but at present we suggest there may be two systems segregating in this species: a ZW system on LG04, and an XY system on LG02.

### Analysis of *Paracyprichromis brieni*

*Paracyprichromis brieni* did not have identifiable sex chromosomes using the k-mer method. The analysis of sex-patterned SNPs in 100 kb windows yielded a significant Kruskal–Wallis test (p = 2.389e−6). The Dunn’s test showed significant differences between LG02 and the other chromosomes (Supplemental Table 6), and LG02 had distinct median rank (Fig. 4). There is no evidence that *P. brieni* has the LG15 XY system confirmed in *P. nigripinnis,* or the LG04 ZW system suggested in *P.* sp. ‘brieni south’. With the current data, we tentatively suggest an XY system on LG02 in this species.

## Discussion

### k-mer analysis of sex chromosomes

As demonstrated by previous studies^22–24^, k-mer analysis is an effective method for sex chromosome identification. It is potentially more sensitive than other currently available methods, though sensitivity can be dependent on many properties of the sex chromosomes themselves. We achieved with k-mer analysis what could not be done with GWAS by identifying the sex chromosome from the genomic data for individual samples sequenced to low coverage. Additionally, a large portion of the k-mer pipeline can be conducted without use of a reference genome. Only at the stage where spatial information is desired is a reference genome needed. This reference-free quality of the k-mer method allows for comparison of k-mers between species without the potential inconsistencies introduced by a reference genome. One can easily determine shared sex-specific k-mers between species without needing a reference. As exemplified by the disagreement surrounding sex chromosome evolution in guppies, the reference genome chosen can have a huge impact on sex chromosome detection and theories of evolutionary mechanisms^47,56^. Methods that can avoid the use of a reference genome may produce more replicable results.

### Sex chromosomes in the *Cyprichromini*

Using our k-mer method we confirmed sex chromosomes previously identified in eight species using association tests on genomic and transcriptomic data^32,37^. We also confirmed the sex chromosomes in five additional species that had previously been called based only on a tribe-wise GWAS analysis^32^.

The k-mer method seems to be sensitive enough to detect all but one previously known sex determining system in the Cyprichromini, the LG15 XY in *P. nigripinnis*. Our difficulty in identifying a definitive sex chromosome for *P. brieni* has several possible explanations. The first is that the sex determining region in this species might be small and/or young. If this is the case, analyzing the segregation of sex in a family would increase linkage disequilibrium and might allow detection of a small/weakly differentiated region. The second possibility is that there are multiple sex determining systems segregating within species. There is precedence for this in several other species of East African cichlids^14,57,58^. Identification of sex chromosomes in this case would require either larger samples of unrelated individuals, or the analysis of segregation in multiple independent families (e.g. Ser^14^). Increasing the size of the male and female pools greatly improves the sensitivity of both the k-mer and the sex-specific SNP methods, as exemplified in our results for *C. leptosoma*. Having such data for *P.* sp. “brieni south” allowed us to identify a very small sex differentiated region.

### Transitions among sex chromosome systems

**Figure 5:**
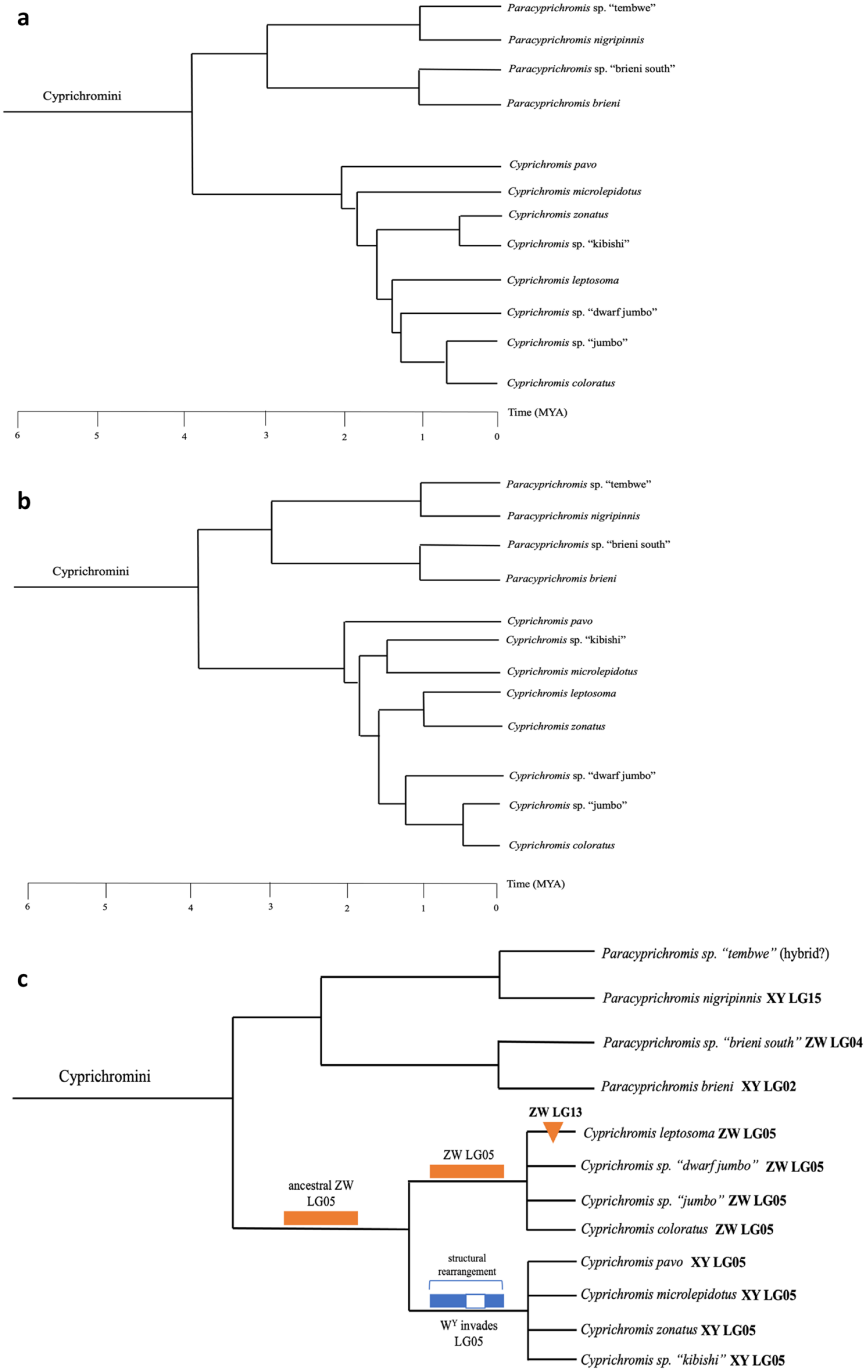
Phylogeny of the *Cyprichromini*. (**a**) RAxML tree and (**b**) SNAPP tree for the Cyprichromini adapted from Ronco et al. 2021. (**c**) Simplified tree to illustrate the transitions in sex determination in the Cyprichromini. The proposed sequence for the sex chromosome turnover, from a ZW system spanning most of LG05 in the ancestral *Cyprichromis*, to an XY system spanning a slightly smaller region of LG05 is shown. An additional evolutionary sex determining region on LG13 has been added to the sex chromosome in *C. leptosoma*. *Paracyprichromis nigripinnis* has an XY system on LG15 which is not found in other species in the genus. A ZW system on LG04 was identified in *P.* sp. ‘brieni south*’,* but no sex locus could be identified in its sister species *P. brieni*. The sample of *P.* sp. “tembwe” likely has a recent hybrid origin.

A primary motivation for our studies of cichlid sex chromosomes is to quantify the rates and patterns of transition from one sex chromosome system to another. Previous work suggested at least two transitions in the tribe Cyprichromini, and an ancestral state of XY on LG05 for the genus *Cyprichromis* (Fig. 5a)^32^. In contrast, we suggest that there have been many more transitions, particularly in *Paracyprichromis*, and that the ancestral state for *Cyprichromis* was ZW on LG05.

Of the 3 species of *Paracyprichromis* studied, only one (*P. nigripinnis*) has a highly differentiated sex chromosome (LG15 XY) at high frequency. *P.* sp. “brieni south” has a narrow ZW signal on LG04, as well as statistically significant evidence for XY differentiation of LG02. For *P*. *brieni*, we found inconclusive evidence for XY differentiation on several chromosomes (LG02, LG06 and LG08). Importantly, we can rule out the presence of an LG15 XY system in our samples of both *P. brieni* and *P*. sp. “brieni south”. The existence of multiple and/or young sex chromosomes in these species indicates a high rate of sex chromosome transition in this group.

Current phylogenies for the Cyprichromini are based on whole genome sequences^36^. The RAxML tree (Fig. 5a) and the SNAPP tree (Fig. 5b) for Lake Tanganyika cichlids reported by these authors are both highly supported, but statistical support for some of the key relationships within the Cyprichromini are not well supported. Previous analyses did not compare the level of differentiation in the LG05 ZW and XY systems^32^. Our analyses demonstrate that the LG05 ZW system is roughly seven times more differentiated, and therefore likely older than the LG05 XY system. We contend that the LG05 ZW system is either ancestral for *Cyprichromis*, or that the XY system was polymorphic as far back as *C. pavo*. Alternatively, the ZW species could have a highly accelerated rate of accumulation of differentiation, relative to the XY species. This scenario seems unlikely, given that the level of ZW differentiation is so much higher than the XY differentiation.

On the RAxML tree, our preferred scenario would require a period of polymorphism in which both the ZW and XY systems were segregating, and at least three convergent fixations of the XY system. On the SNAPP topology, the period of polymorphism would be longer, and at least three convergent fixations of the XY system would again be required. The simplest alternative tree (Fig. 5c) would require a single transition from ZW to XY, and would eliminate convergence, but is incompatible with any phylogenetic tree hitherto published. The four ZW species have identical regions of ZW divergence on LG05. There is an additional ZW sex determining region on LG13 unique to *C. leptosoma*.

Regardless of how the transition played out during the radiation of *Cyprichromis*, it is interesting to consider how the heterogeneous *cis* transition from a ZW to an XY system may have taken place. We propose that the new Y arose on a W chromosome (W^Y^). As the frequency of individuals with W^Y^ increased in the population, there would be a corresponding increase in the frequency of WW females. Eventually the Z chromosome was lost, resulting in a WW/WW^Y^, or more simply an XX/XY, sex-determining system.

This scenario is seemingly inconsistent with current theory for sex chromosome transitions in two respects. First, the W chromosome should be a hostile environment for the evolution of a new Y chromosome because of linked sexually antagonistic variation favoring females. Second, WW females should suffer significant fitness consequences arising from homozygosity of deleterious alleles, inhibiting an increase in the frequency of the W^7,59^. We have no insights into how the W^Y^ was able to invade, despite the likely presence of linked alleles favoring females, except to postulate that the W^Y^ was nevertheless more fit than the Z.

With respect to mutational load in WW females we note that recombination would be restored on the W in homozygotes (both WW and WW^Y^), perhaps allowing purging of some deleterious variants. This purging might have been enhanced by occasional interspecific hybridization among the several XY species. The Y differentiated region spans a smaller region of LG05. Recombination between the W^Y^ and W occurs across the chromosome, except for the new Y-specific region (a new structural variant?). Thus, the pseudoautosomal region is larger in the XY than in the ZW system.

### Divergence of sex chromosomes

Highly divergent sex chromosomes have been proposed to develop in a stepwise manner, as recombination is restricted gradually over increasing regions of the chromosome^4,59,60^. In contrast, here we show two cases (LG05-ZW and LG05-XY) in which the sex chromosomes are uniformly divergent over most of their length. Instead of the region of differentiation/linkage disequilibrium spreading gradually by stepwise recruitment of other sexually antagonistic genes, it appears that this differentiation occurred in a single step.

The *Cyprichromis* with the ZW system show a consistent level of differentiation across the entire sex determining region on LG05. *C. leptosoma* has a larger number of sex-patterned SNPs than either *C.* sp. “dwarf jumbo” or *C. coloratus*. *C. leptosoma* has signal for younger ZW strata on LG13 and LG03 (apparent in both pool-seq and single individual data), as well as some ZW signal on LG11. These additional signals are not shared with the other LG05 ZW species. The larger range of *C. leptosoma* suggests it may have a larger population size, which would make selection more effective, allowing accumulation of even weakly selected sexually antagonistic alleles.

After the transition to the LG05 XY system, *C. pavo* appears to have diverged more rapidly than the other three species. *C. pavo* and *C. zonatus* have stronger signal than *C. microlepidotus* and *C.* sp. “kibishi”*.* There is little apparent change to the regions of the chromosome that appear differentiated between these four, so the differences must occur within the core region. Very little expansion of the differentiated regions has occurred in *C. pavo*, suggested to be the most ancestral species in the sub-clade by El Taher et al.^36^, compared to that in *C.* sp. “kibishi”. The rapid establishment and conserved boundaries of the differentiated region may be the result of one or more structural rearrangements during the establishment of the Y chromosome. This structural arrangement did not include the region from 20.7 to 26.2 Mb on the Y, which continues to recombine with the X. Further analysis of the structure of LG05 will require long-read sequencing.

### Limited options?

The “limited options” hypothesis suggests that some genes may be better suited to becoming sex determiners, or that some chromosomes may be better suited to becoming sex chromosomes^61^. A non-random pattern of sex chromosome recruitment in Lake Tanganyika cichlids has been claimed to support the ‘limited options’ hypothesis^32^. This claim seems premature, given that sex chromosomes have been identified on at least 14 of 22 linkage groups among just the Lake Tanganyika species, that the sex chromosome systems of many Tanganyika species still have not been identified, and that the causative genes for sex determination have not been identified for any species from Lake Tanganyika.

It is true that LG05 has been recruited as a sex chromosome several times during the radiation of cichlids in East Africa. In Lake Malawi, LG05 has been recruited as a sex chromosome as a ZW system heavily influenced by sexual conflict^15,62^. In *Astatotilapia burtoni*, which displays polygenic sex determination, LG05 has been recruited as an XY system in a fusion with LG14^51,63^. Considering the size of the sex determining region on LG05 in *Cyprichromis*, it is not surprising that it overlaps with the LG05 system in these other species. However, until we are able to identify the sex determining gene in each of these species, we cannot determine conclusively if this represents convergent evolution consistent with the strict (genic) ‘limited options’ hypothesis.

### Conclusions

Many commonly held beliefs about the evolution of sex chromosomes have not been tested because of a lack of data on recent sex chromosome transitions. By examining the evolution of sex chromosomes among closely related species of *Cyprichromini*, we present new insight into how heterogeneous *cis* transitions occur, an area of sex chromosome biology that is not well studied. Additionally, we document the rapid development of two large sex determining regions with little subsequent expansion, contradicting the theory of progressive expansion of sex determining regions. This study adds to the existing body of research on the frequent transitions among sex chromosomes in cichlids. These transitions involve at least 12 of 22 chromosomes^11^ and potentially include both heterogeneous and homogeneous transitions on the same and different chromosomes.

## Supplementary Information

Supplementary Information.

## Data Availability

The newly sequenced data for *Paracyprichromis* sp. “brieni south” pool-seq are available on NCBI under BioProject: PRJNA802233. Previously published data are available under their respective BioProjects on NCBI, Gammerdinger et al. 2018 BioProject: PRJNA400462, Ronco et al. 2020 BioProject: PRJNA550295, and El Taher et al. 2021 BioProject: PRJNA552202.

## Code availability

All previously unreported custom source code is available at https://github.com/KristenBehrens/K-mer-scripts.
