# Supplementary material for: Sex chromosomes in the tribe Cyprichromini (Teleostei: Cichlidae) of Lake Tanganyika
Source: Sci Rep. 2022 Oct 26;12:17998. doi: 10.1038/s41598-022-23017-y (PMC9606112; doi:10.1038/s41598-022-23017-y)
Supplement: Supplementary file 1 — Supplementary Information. [file 41598_2022_23017_MOESM1_ESM.pdf]

Supplementary Tables and Figures

Sex Chromosomes in the Tribe Cyprichromini (Teleostei: Cichlidae) of Lake Tanganyika

Kristen A. Behrens [1], Stephan Koblmüller [2], Thomas D. Kocher [1]

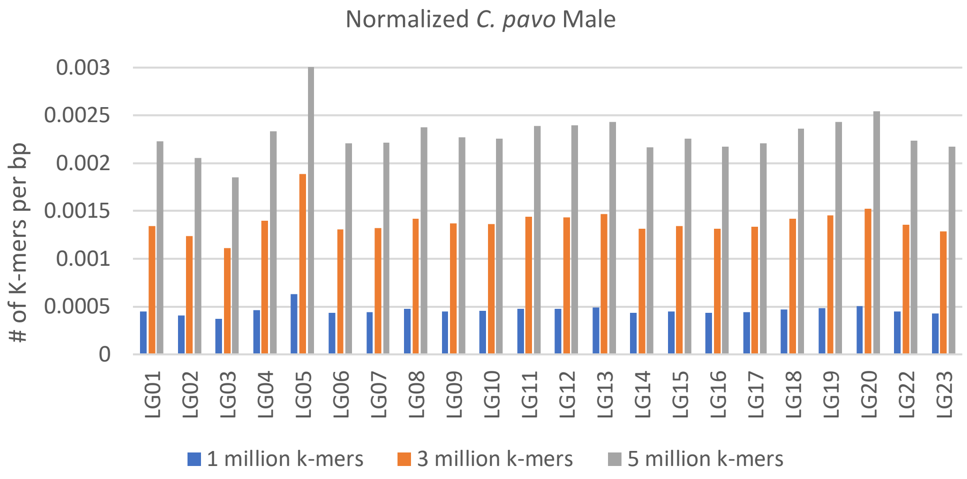

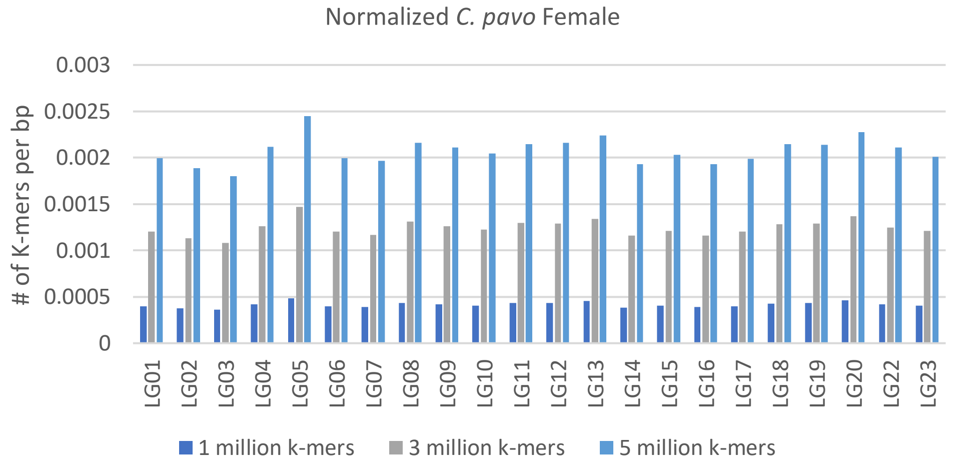

Supplemental Figure 1. Validation of 1 million k-mer subset for assignment of position.

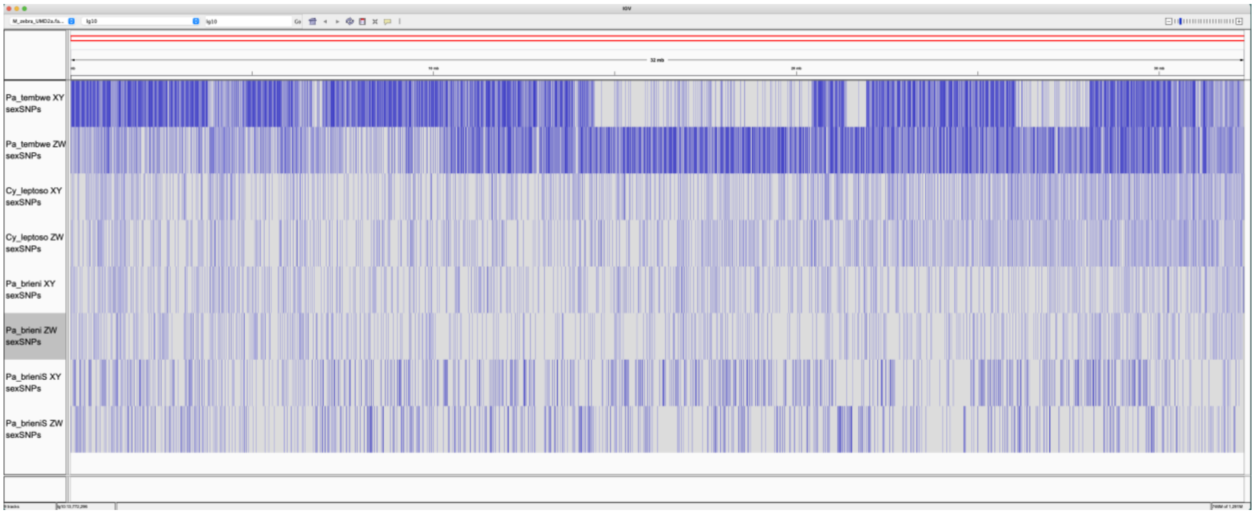

Supplemental Figure 2. *P. sp. "tembwe"* hybrids

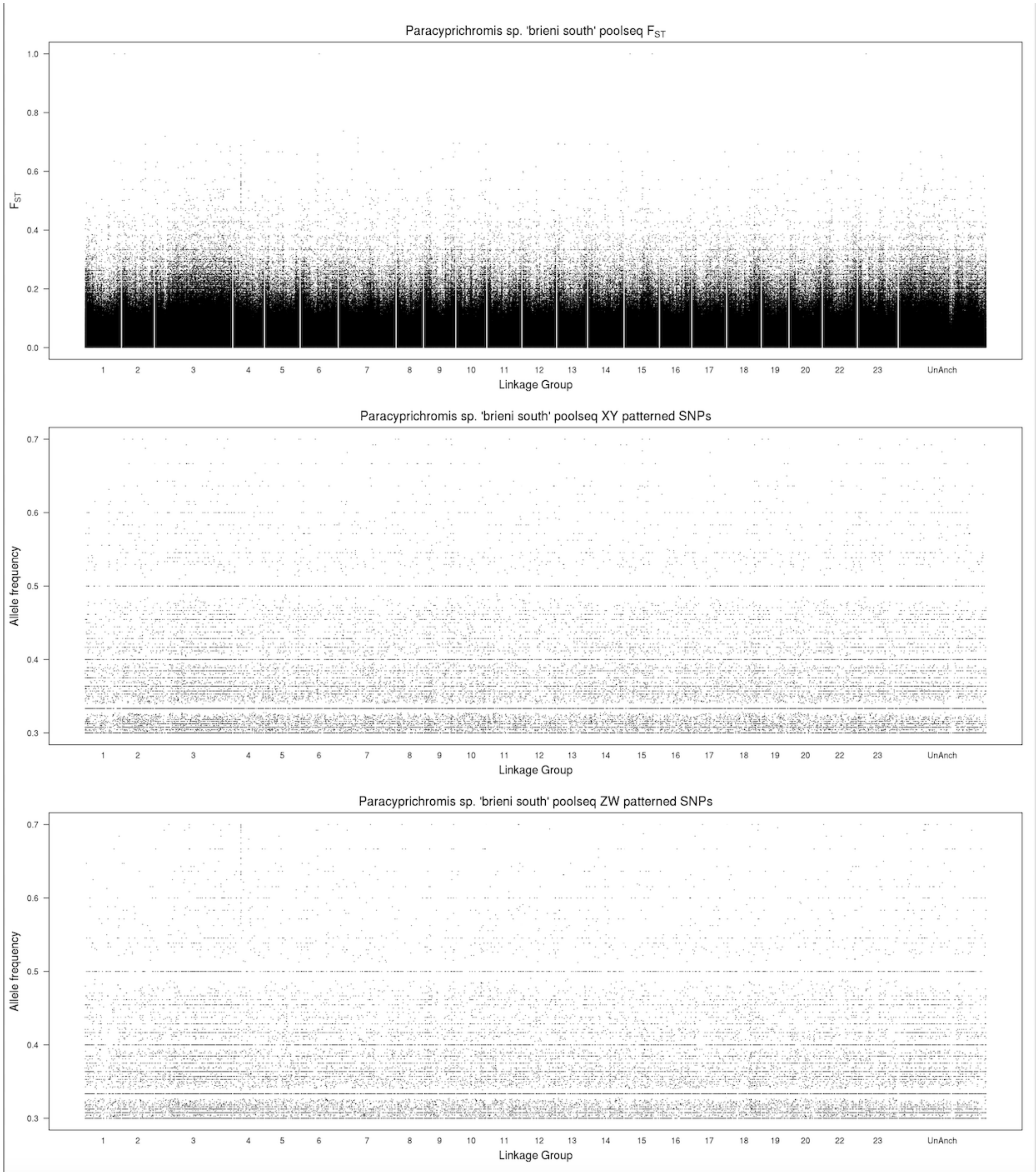

Supplemental Figure 3. Whole genome F_{ST} (top panel), XY-patterned SNPs (middle panel), and ZW-patterned SNPs (bottom panel) for pool-seq *Paracyprichromis* sp. brieni south.

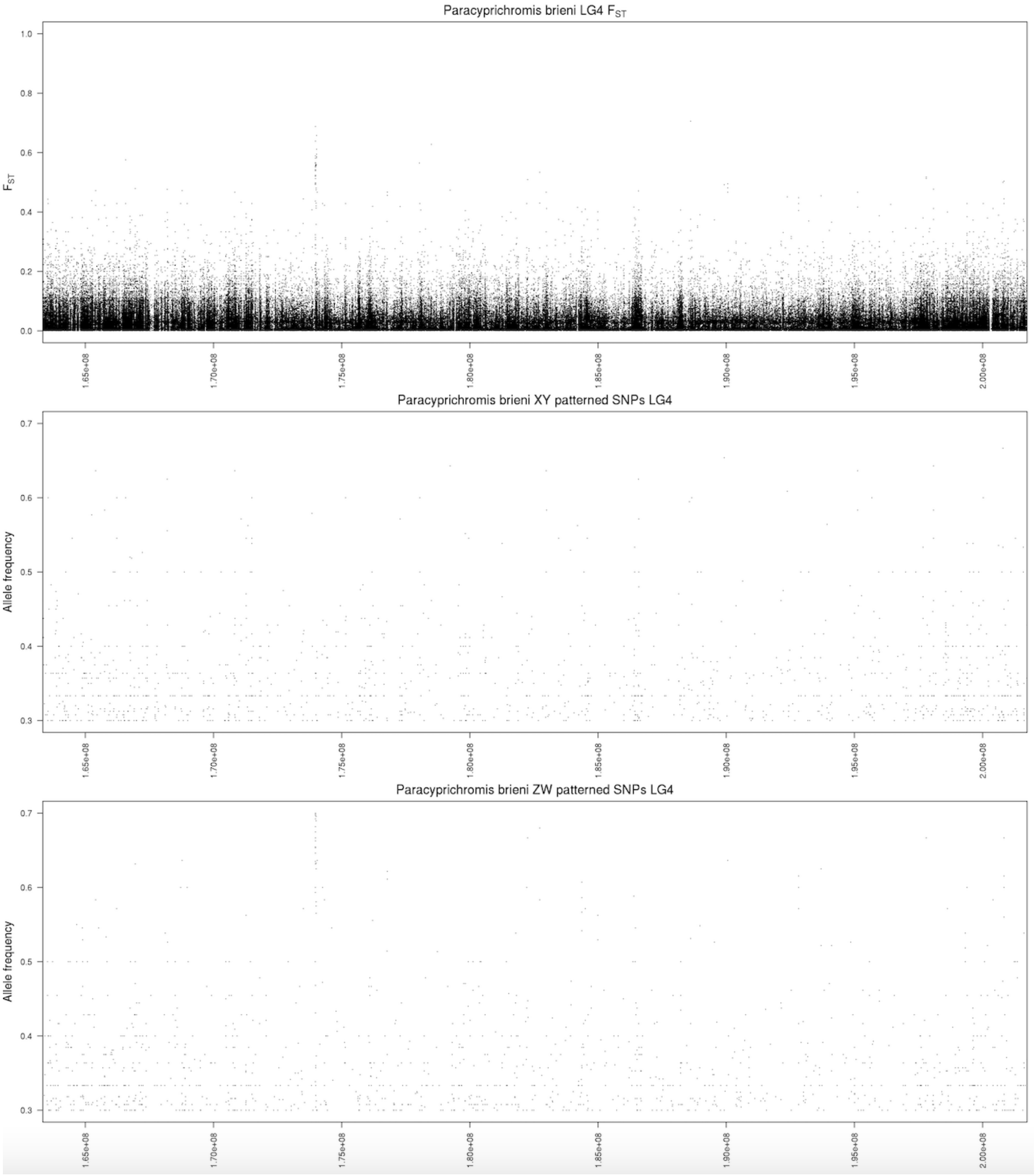

Supplemental Figure 4. Single chromosome plot of pool-seq *Paracyprichromis* sp. Brieni south F_{ST} and sex patterned SNPs.

Supplemental Table 1. *C. leptosoma* Dunn's Test Reults, pooledseq (UMD2a)

	LG1	LG2	LG3	LG4	LG5	LG6	LG7	LG8	LG9	LG10	LG11	LG12	LG13	LG14	LG15	LG16	LG17	LG18	LG19	LG20	LG22	LG23
**LG1**	-																					
**LG2**	ns	-																				
**LG3**	****	****	-																			
**LG4**	ns	*	****	-																		
**LG5**	****	****	****	****	-																	
**LG6**	*	ns	****	****	****	-																
**LG7**	ns	ns	****	ns	****	***	-															
**LG8**	ns	ns	****	**	****	ns	*	-														
**LG9**	ns	ns	****	***	****	ns	**	ns	-													
**LG10**	ns	ns	****	ns	****	*	ns	ns	ns	-												
**LG11**	****	**	****	****	****	ns	****	*	ns	***	-											
**LG12**	ns	ns	****	ns	****	ns	ns	ns	ns	ns	***	-										
**LG13**	****	****	****	****	****	****	****	****	****	****	****	****	-									
**LG14**	ns	ns	****	ns	****	**	ns	ns	*	ns	****	ns	****	-								
**LG15**	ns	*	****	ns	****	****	ns	**	**	ns	****	ns	****	ns	-							
**LG16**	***	*	****	****	****	ns	****	ns	ns	**	ns	**	****	****	****	-						
**LG17**	ns	ns	****	**	****	ns	*	ns	ns	ns	*	ns	****	ns	**	ns	-					
**LG18**	*	ns	****	****	****	ns	***	ns	ns	*	ns	*	****	**	****	ns	ns	-				
**LG19**	ns	ns	****	*	****	ns	ns	ns	ns	ns	*	ns	****	ns	*	*	ns	ns	-			
**LG20**	*	ns	****	***	****	ns	**	ns	ns	ns	ns	ns	****	**	***	ns	ns	ns	ns	-		
**LG22**	****	****	***	****	****	*	****	**	*	****	ns	****	****	****	****	ns	**	ns	**	*	-	
**LG23**	ns	ns	****	ns	****	*	ns	ns	ns	ns	****	ns	****	ns	ns	***	ns	*	ns	*	****	-

Supplemental Table 2. *C. leptosoma* Dunn's Test Results, single individual (UMD2a)

	LG1	LG2	LG3	LG4	LG5	LG6	LG7	LG8	LG9	LG10	LG11	LG12	LG13	LG14	LG15	LG16	LG17	LG18	LG19	LG20	LG22	LG23
**LG1**	-																					
**LG2**	ns	-																				
**LG3**	ns	ns	-																			
**LG4**	****	****	****	-																		
**LG5**	****	****	****	****	-																	
**LG6**	*	***	*	*	****	-																
**LG7**	****	****	****	ns	****	*	-															
**LG8**	ns	ns	ns	**	****	ns	***	-														
**LG9**	ns	ns	ns	****	****	*	****	ns	-													
**LG10**	ns	**	*	*	****	ns	*	ns	*	-												
**LG11**	ns	***	*	*	****	ns	*	ns	*	ns	-											
**LG12**	ns	ns	ns	****	****	**	****	ns	ns	*	*	-										
**LG13**	****	****	****	****	****	****	****	****	****	****	****	****	-									
**LG14**	***	****	****	ns	****	ns	ns	**	****	ns	ns	****	****	-								
**LG15**	**	****	**	ns	****	ns	ns	ns	**	ns	ns	***	****	ns	-							
**LG16**	ns	ns	ns	****	****	**	****	ns	ns	ns	ns	ns	****	****	**	-						
**LG17**	*	****	**	ns	****	ns	ns	ns	**	ns	ns	**	****	ns	ns	*	-					
**LG18**	**	ns	*	****	****	****	****	**	ns	****	****	ns	**	****	****	*	****	-				
**LG19**	ns	ns	ns	****	****	**	****	ns	ns	*	*	ns	****	****	***	ns	**	ns	-			
**LG20**	**	ns	*	****	****	****	****	**	ns	****	****	ns	**	****	****	*	****	ns	ns	-		
**LG22**	ns	ns	ns	***	****	ns	****	ns	ns	ns	ns	ns	****	***	*	ns	ns	**	ns	**	-	
**LG23**	**	****	***	ns	****	ns	ns	*	***	ns	ns	***	****	ns	ns	**	ns	****	***	****	**	-

Supplemental Table 3. *P.* sp. "brieni south" Dunn*'s Test results, pooled (UMD2a)*

	LG1	LG2	LG3	LG4	LG5	LG6	LG7	LG8	LG9	LG10	LG11	LG12	LG13	LG14	LG15	LG16	LG17	LG18	LG19	LG20	LG22	LG23
**LG1**	-																					
**LG2**	*	-																				
**LG3**	ns	**	-																			
**LG4**	ns	*	ns	-																		
**LG5**	ns	ns	ns	ns	-																	
**LG6**	ns	*	ns	ns	ns	-																
**LG7**	ns	*	ns	ns	ns	ns	-															
**LG8**	ns	ns	ns	ns	ns	ns	ns	-														
**LG9**	ns	*	ns	ns	ns	ns	ns	ns	-													
**LG10**	ns	*	ns	ns	ns	ns	ns	ns	ns	-												
**LG11**	ns	ns	ns	ns	ns	ns	ns	ns	ns	ns	-											
**LG12**	ns	*	ns	ns	ns	ns	ns	ns	ns	ns	ns	-										
**LG13**	ns	**	ns	ns	ns	ns	ns	ns	ns	ns	ns	ns	-									
**LG14**	ns	**	ns	ns	ns	ns	ns	ns	ns	ns	ns	ns	ns	-								
**LG15**	ns	ns	ns	ns	ns	ns	ns	ns	ns	ns	ns	ns	ns	ns	-							
**LG16**	ns	*	ns	ns	ns	ns	ns	ns	ns	ns	ns	ns	ns	ns	ns	-						
**LG17**	ns	*	ns	ns	ns	ns	ns	ns	ns	ns	ns	ns	ns	ns	ns	ns	-					
**LG18**	ns	***	ns	ns	ns	ns	ns	ns	ns	ns	ns	ns	ns	ns	*	ns	ns	-				
**LG19**	ns	ns	ns	ns	ns	ns	ns	ns	ns	ns	ns	ns	ns	ns	ns	ns	ns	ns	-			
**LG20**	ns	ns	ns	ns	ns	ns	ns	ns	ns	ns	ns	ns	ns	ns	ns	ns	ns	ns	ns	-		
**LG22**	ns	****	ns	ns	ns	ns	ns	ns	ns	ns	ns	ns	ns	ns	**	ns	ns	ns	ns	ns	-	
**LG23**	ns	**	ns	ns	ns	ns	ns	ns	ns	ns	ns	ns	ns	ns	ns	ns	ns	ns	ns	ns	ns	-

Supplemental Table 4. *P.* sp. "brieni south" Dunn's Test results, pooled (UMDNMBU)

	LG1	LG2	LG3	LG4	LG5	LG6	LG7	LG8	LG9	LG10	LG11	LG12	LG13	LG14	LG15	LG16	LG17	LG18	LG19	LG20	LG22	LG23
**LG1**	-																					
**LG2**	*	-																				
**LG3**	ns	**	-																			
**LG4**	ns	**	ns	-																		
**LG5**	ns	ns	ns	ns	-																	
**LG6**	ns	**	ns	ns	ns	-																
**LG7**	ns	ns	ns	ns	ns	ns	-															
**LG8**	ns	ns	ns	ns	ns	ns	ns	-														
**LG9**	ns	ns	ns	ns	ns	ns	ns	ns	-													
**LG10**	ns	ns	ns	ns	ns	ns	ns	ns	ns	-												
**LG11**	ns	***	ns	ns	*	ns	ns	ns	ns	ns	-											
**LG12**	ns	**	ns	ns	ns	ns	ns	ns	ns	ns	ns	-										
**LG13**	ns	**	ns	ns	ns	ns	ns	ns	ns	ns	ns	ns	-									
**LG14**	ns	*	ns	ns	ns	ns	ns	ns	ns	ns	ns	ns	ns	-								
**LG15**	ns	ns	ns	ns	ns	ns	ns	ns	ns	ns	*	ns	ns	ns	-							
**LG16**	ns	ns	ns	ns	ns	ns	ns	ns	ns	ns	ns	ns	ns	ns	ns	-						
**LG17**	ns	*	ns	ns	ns	ns	ns	ns	ns	ns	ns	ns	ns	ns	ns	ns	-					
**LG18**	ns	**	ns	ns	ns	ns	ns	ns	ns	ns	ns	ns	ns	ns	ns	ns	ns	-				
**LG19**	ns	ns	ns	ns	ns	ns	ns	ns	ns	ns	ns	ns	ns	ns	ns	ns	ns	ns	-			
**LG20**	ns	**	ns	ns	ns	ns	ns	ns	ns	ns	ns	ns	ns	ns	ns	ns	ns	ns	ns	-		
**LG22**	ns	**	ns	ns	ns	ns	ns	ns	ns	ns	ns	ns	ns	ns	ns	ns	ns	ns	ns	ns	-	
**LG23**	ns	ns	ns	ns	ns	ns	ns	ns	ns	ns	ns	ns	ns	ns	ns	ns	ns	ns	ns	ns	ns	-

Supplemental Table 5. *P.* sp. "brieni south" Dunn's Test results, single individual (UMD2a)

	**LG1**	**LG2**	**LG3**	**LG4**	**LG5**	**LG6**	**LG7**	**LG8**	**LG9**	**LG10**	**LG11**	**LG12**	**LG13**	**LG14**	**LG15**	**LG16**	**LG17**	**LG18**	**LG19**	**LG20**	**LG22**	**LG23**
**LG1**	-																					
**LG2**	*	-																				
**LG3**	ns	**	-																			
**LG4**	ns	ns	ns	-																		
**LG5**	ns	ns	**	ns	-																	
**LG6**	ns	***	ns	*	***	-																
**LG7**	ns	*	ns	ns	ns	*	-															
**LG8**	**	ns	***	ns	ns	****	**	-														
**LG9**	ns	ns	ns	ns	ns	ns	ns	ns	-													
**LG10**	ns	ns	ns	ns	ns	ns	ns	*	ns	-												
**LG11**	ns	*	ns	ns	ns	ns	ns	*	ns	ns	-											
**LG12**	ns	***	ns	ns	**	ns	ns	***	ns	ns	ns	-										
**LG13**	ns	ns	ns	ns	ns	*	ns	ns	ns	ns	ns	ns	-									
**LG14**	ns	**	ns	ns	*	ns	ns	**	ns	ns	ns	ns	ns	-								
**LG15**	ns	**	ns	ns	*	ns	ns	**	ns	ns	ns	ns	ns	ns	-							
**LG16**	ns	ns	*	ns	ns	**	ns	ns	ns	ns	ns	**	ns	ns	ns	-						
**LG17**	ns	*	ns	ns	ns	ns	nsn	*	ns	ns	ns	ns	ns	ns	ns	ns	-					
**LG18**	ns	ns	**	ns	ns	***	ns	ns	ns	ns	ns	**	ns	*	*	ns	ns	-				
**LG19**	ns	ns	ns	ns	ns	**	ns	ns	ns	ns	ns	*	ns	ns	ns	ns	ns	ns	-			
**LG20**	**	ns	***	ns	ns	****	**	ns	ns	*	**	***	ns	**	***	ns	**	ns	ns	-		
**LG22**	ns	ns	ns	ns	ns	*	ns	*	ns	ns	ns	ns	ns	ns	ns	ns	ns	ns	ns	*	-	
**LG23**	ns	***	ns	ns	**	ns	ns	***	ns	ns	ns	ns	ns	ns	ns	*	ns	**	*	***	ns	-

Supplemental Table 6. *P. brieni* Dunn's Test results, single individual (UMD2a)

	LG1	LG2	LG3	LG4	LG5	LG6	LG7	LG8	LG9	LG10	LG11	LG12	LG13	LG14	LG15	LG16	LG17	LG18	LG19	LG20	LG22	LG23
**LG1**	-																					
**LG2**	*	-																				
**LG3**	ns	**	-																			
**LG4**	ns	*	ns	-																		
**LG5**	ns	ns	ns	ns	-																	
**LG6**	ns	*	ns	ns	ns	-																
**LG7**	ns	*	ns	ns	ns	ns	-															
**LG8**	ns	ns	ns	ns	ns	ns	ns	-														
**LG9**	ns	*	ns	ns	ns	ns	ns	ns	-													
**LG10**	ns	*	ns	ns	ns	ns	ns	ns	ns	-												
**LG11**	ns	ns	ns	ns	ns	ns	ns	ns	ns	ns	-											
**LG12**	ns	*	ns	ns	ns	ns	ns	ns	ns	ns	ns	-										
**LG13**	ns	**	ns	ns	ns	ns	ns	ns	ns	ns	ns	ns	-									
**LG14**	ns	**	ns	ns	ns	ns	ns	ns	ns	ns	ns	ns	ns	-								
**LG15**	ns	ns	ns	ns	ns	ns	ns	ns	ns	ns	ns	ns	ns	ns	-							
**LG16**	ns	*	ns	ns	ns	ns	ns	ns	ns	ns	ns	ns	ns	ns	ns	-						
**LG17**	ns	*	ns	ns	ns	ns	ns	ns	ns	ns	ns	ns	ns	ns	ns	ns	-					
**LG18**	ns	***	ns	ns	ns	ns	ns	ns	ns	ns	ns	ns	ns	ns	*	ns	ns	-				
**LG19**	ns	ns	ns	ns	ns	ns	ns	ns	ns	ns	ns	ns	ns	ns	ns	ns	ns	ns	-			
**LG20**	ns	*	ns	ns	ns	ns	ns	ns	ns	ns	ns	ns	ns	ns	ns	ns	ns	ns	ns	-		
**LG22**	ns	****	ns	ns	ns	ns	ns	ns	ns	ns	ns	ns	ns	ns	**	ns	ns	ns	ns	ns	-	
**LG23**	ns	**	ns	ns	ns	ns	ns	ns	ns	ns	ns	ns	ns	ns	ns	ns	ns	ns	ns	ns	ns	-

Supplemental Table 7. *P. nigripinnis* Dunn's Test results, single individual (UMD2a)

	LG1	LG2	LG3	LG4	LG5	LG6	LG7	LG8	LG9	LG10	LG11	LG12	LG13	LG14	LG15	LG16	LG17	LG18	LG19	LG20	LG22	LG23
**LG1**	-																					
**LG2**	ns	-																				
**LG3**	ns	ns	-																			
**LG4**	ns	ns	ns	-																		
**LG5**	ns	ns	ns	ns	-																	
**LG6**	**	***	***	*	ns	-																
**LG7**	*	**	***	ns	ns	ns	-															
**LG8**	ns	ns	ns	ns	ns	*	ns	-														
**LG9**	ns	ns	ns	ns	*	****	***	ns	-													
**LG10**	ns	ns	ns	ns	ns	**	**	ns	ns	-												
**LG11**	*	ns	ns	*	**	****	****	*	ns	ns	-											
**LG12**	ns	ns	ns	ns	*	****	***	ns	ns	ns	ns	-										
**LG13**	ns	ns	ns	ns	ns	***	**	ns	ns	ns	ns	ns	-									
**LG14**	ns	ns	ns	ns	ns	ns	ns	ns	*	ns	**	*	ns	-								
**LG15**	****	****	****	****	****	****	****	****	****	****	****	****	****	****	-							
**LG16**	ns	ns	ns	ns	ns	**	*	ns	ns	ns	ns	ns	ns	ns	****	-						
**LG17**	ns	ns	ns	ns	ns	***	**	ns	ns	ns	ns	ns	ns	ns	****	ns	-					
**LG18**	ns	ns	ns	ns	ns	**	*	ns	ns	ns	ns	ns	ns	ns	****	ns	ns	-				
**LG19**	***	****	****	***	**	ns	ns	**	****	****	****	****	****	**	****	****	****	****	-			
**LG20**	*	**	**	ns	ns	ns	ns	ns	***	*	****	***	**	ns	****	*	ns	ns	ns	-		
**LG22**	ns	ns	ns	ns	*	****	****	ns	ns	ns	ns	ns	ns	*	****	ns	ns	ns	****	***	-	
**LG23**	*	ns	ns	*	***	****	****	*	ns	ns	ns	ns	ns	***	****	*	ns	ns	****	****	ns	-
